# Effects of coenzyme Q10 on ovarian surface epithelium-derived ovarian stem cells and ovarian function in a 4-vinylcyclohexene diepoxide-induced murine model of ovarian failure

**DOI:** 10.1186/s12958-021-00736-x

**Published:** 2021-04-22

**Authors:** Hyun Joo Lee, Min Jung Park, Bo Sun Joo, Jong Kil Joo, Yeon Hee Kim, Sun Woo Yang, Chang-Woon Kim, Ki Hyung Kim

**Affiliations:** 1 Department of Obstetrics and Gynecology, Pusan National University School of Medicine, Busan, Republic of Korea; 2 Biomedical Research Institute, Pusan National University Hospital, Busan, 49241 Republic of Korea; 3 The Korea Institute for Public Sperm Bank, Busan, Republic of Korea; 4 Department of Obstetrics and Gynecology, Samsung Changwon Hospital, Sungkyunkwan University School of Medicine, Changwoon, Kyungsang Nam-Do Republic of Korea

**Keywords:** CoQ10, Ovarian failure, Ovarian stem cells, Ovarian function

## Abstract

**Background:**

Several studies have shown that coenzyme Q10 (CoQ10) can rescue ovarian aging and that ovarian surface epithelium (OSE)-derived ovarian stem cells (OSCs) are useful for treating infertility due to ovarian aging. However, few studies have examined the effect of CoQ10 on OSCs. This study was aimed to investigate whether CoQ10 activates OSCs and recovers ovarian function in a 4-vinylcyclohexene diepoxide (VCD)-induced mouse model of ovarian failure.

**Methods:**

Forty female C57BL/6 mice aged 6 weeks were randomly divided into four groups (*n* = 10/group): a control group administered saline orally, a CoQ10 group administered 150 mg/kg/day of CoQ10 orally in 1 mL of saline daily for 14 days, a VCD group administered 160 mg/kg/day of VCD i.p. in 2.5 mL of saline/kg for 5 days, and a VCD + CoQ10 group administered VCD i.p. for 5 days injection and CoQ10 (150 mg/kg/day) orally for 14 days. After treatment, follicle counts were evaluated by hematoxylin and eosin (H&E) staining, and ovarian mRNA expressions of Bmp-15, Gdf-9, and c-Kit were examined by quantitative real-time PCR. Serum FSH, AMH, and ROS levels were also measured. Oocyte-like structure counts and the expressions of Oct-4 and MVH were also evaluated after culturing OSE for 3 weeks. In a second experiment, 32 female mice were administered CoQ10 as described above, induced to superovulate using PMSG and hCG, and mated. Numbers of zygotes and embryo development rate were examined.

**Results:**

Postcultured OSE showed significant increases in the numbers of oocyte-like structure and that the expression of Oct-4 and MVH were higher in the VCD + CoQ10 group than in the VCD group (*p <* 0.05). Numbers of surviving follicles from primordial to antral follicles, numbers of zygotes retrieved and embryo development rate to blastocyst were significantly greater in the VCD + CoQ10 group than in the VCD group (*p <* 0.01). Serum AMH level and ovarian expressions of Bmp-15, Gdf-9 and c-Kit were also significantly greater in the VCD + CoQ10 group than in the VCD group (*p <* 0.05). In contrast, serum ROS level was significantly lower in the VCD + CoQ10 group than in the VCD group (*p <* 0.05).

**Conclusion:**

This study shows that CoQ10 stimulates the differentiation of OSE-derived OSCs and confirms that CoQ10 can reduce ROS levels and improve ovarian function and oocyte quality in mice with VCD-induced ovarian failure.

## Content

Coenzyme Q10 stimulates the differentiation of ovarian surface epithelium-derived ovarian stem cells and recovers ovarian function in mice with VCD-induced ovarian failure.

## Background

Ovarian aging remains as an unmet medical need in the context of female infertility, and the difficulties of treating ovarian aging is primarily due to a severe deterioration of oocyte quality. The main cause of age-related decline in oocyte quality is known to be associated with mitochondrial dysfunction in oocytes, which leads to oxidative stress (OS) [1]. OS is caused by an excessive generation of reactive oxygen species (ROS), which are highly unstable and reactive and can easily damage the basic tissue components and disrupt various cellular events and functions of major biomolecules such as proteins, lipids, and DNA [2]. Due to these characteristics, OS induces cellular senescence, diminishes ovarian reserves and oocyte quality, and ultimately causes follicular atresia [3]. Accordingly, antioxidants have been viewed as an important means of preventing OS-induced ovarian aging by improving mitochondrial function [4–6].

Coenzyme Q10 (CoQ10) is one of the representative antioxidants [7, 8], which is synthesized by most normal tissues and retards aging [9–11]. Previous reports have shown that oral intake of CoQ10 increases its level in various tissues including muscle, adrenal gland, sperm and ovaries. Furthermore, CoQ10 is also present in human follicular fluid, which suggests it might act as antioxidant in the human reproductive system [12–14]. In fact, several recent studies have shown that CoQ10 can improve mitochondrial function and rescue ovarian aging by protecting ovarian reserve against oxidative damage [15–17].

Recently, many studies have examined the efficacies of stem cell therapies for treating infertility due to ovarian aging. The stem cells include bone marrow-derived mesenchymal stem cells (BMMSCs), adipose-derived mesenchymal stem cells (ADSCS), peripheral blood mononuclear cells (PBMCs), human amniotic epithelial cells (hAECs), amniotic fluid stem cells (AFSCs), umbilical cord mesenchymal stem cells (UCMSCs), menstrual blood-derived stromal cells (MenSCs), and oogonial stem cells [18–20]. Furthermore, studies have demonstrated that ovarian surface epithelium (OSE)-derived ovarian stem cells (OSCs) are present in the ovaries of juvenile and adult mice and in those of postmenopausal women [21–24]. These OSCs can regenerate as normal oocytes if exposed to an appropriate environment such as the young ovary [25, 26], which suggests they might be useful for treating infertility due to ovarian aging, poor response, and premature ovarian insufficiency (POI) [27–29].

However, few studies have investigated the effect of CoQ10 on OSCs and this study was undertaken to investigate whether CoQ10 activates ovarian function and OSCs using a 4-vinylcyclohexene diepoxide (VCD)-induced mouse model of ovarian failure.

## Methods

### Animals

Six-week-old C57BL/6 female mice (body weight: 18.0 ± 2.0 g) were purchased from the Korea Experimental Animal Center (Daegu, Korea) and acclimated in a controlled laboratory (22 ± 2 °C, relative humidity of 55 ± 5% under 12 h light/−dark cycle) for 1 week. Animals were housed in plastic cages and had free access to food pellets and water throughout the study period. The study was designed as a controlled experimental study using laboratory animals. All animal experiments were conducted in accord with the Care and Use of Laboratory Animals protocol issued by the Pusan National University Hospital Institutional Animal Care and Use Committee (PNUH-2020-161).

### Experimental design

Forty female C57BL/6 mice aged 6 weeks were randomly divided into four groups (*n* = 10/group): control group animals were administered with 1 mL of saline orally for 14 days; animals in the CoQ10 group were administered 150 mg/kg/day of CoQ10 orally in 1 mL of saline daily for 14 days; animals in the VCD group were administered 160 mg/kg/day of VCD intraperitoneally (i.p.) at 2.5 mL/kg for 5 days and then 1 mL of oral saline daily for 14 days; and animals in the VCD + CoQ10 group were administered VCD i.p. for 5 days and CoQ10 at 150 mg/kg/day orally for 14 days from the day after VCD administration. VCD was purchased from Sigma-Aldrich Inc. (St. Louis, MO, USA), dissolved in sesame oil (Sigma-Aldrich), and injected with vehicle or 160 mg/kg of VCD (final volume of 2.5 ml/kg) i.p. daily for 5 days.

### Collection of serum and both ovaries

After the final administration of CoQ10 or saline on experimental day (ED) 14, mice were anesthetized with the use of carbon dioxide (CO_2_). Blood samples were taken from heart and collected in plain tube. To induce complete clot formation, whole blood samples were kept at room temperature for 90 min. They were centrifuged at 3000 rpm and 4 °C for 15 min to separate cellular components and serum. After blood collection, both ovaries were removed and weighed. Right ovaries were used for OSC culture, and left ovaries were subjected to histological examination and mRNA analysis.

### Scraping of ovarian surface epithelium (OSE) and in vitro culture of putative ovarian stem cells (OSCs)

Right ovaries were gently rinsed several times in Dulbecco’s phosphate-buffered saline (DPBS; Invitrogen, Carlsbad, CA, USA) containing antibiotics (penicillin 100 U/mL, streptomycin 100 mg/mL; Invitrogen) at ambient temperature and kept in serum-free plain and pre-incubated Dulbecco’s Modified Eagle Medium/Ham’s Nutrient Mixture F-12 (DMEM/F12; Gibco BRL, Grand Island, NY, USA) before OSE was removed by scraping. The surfaces of the remaining intact ovaries were gently scraped several times under an aseptic laminar flow hood using a sterile disposable surgical scalpel (Swann-Morton, Sheffield, United Kingdom) into plain DMEM/F12 in a 60 mm dish at 37 °C on a preheated tray. OSE was easily detached from ovary surfaces and centrifuged to retrieve a scraped suspension of cells. The suspension of scraped OSE cells were transferred to a 15 mL centrifuge tubes and spun at 1000 g for 10 min at room temperature. Pellet was suspended in fresh medium and cultured in DMEM/F12 supplemented with 20% fetal bovine serum (FBS; Invitrogen) and antibiotics (Invitrogen) in a 5% CO_2_ incubator at 37 °C for 3 weeks. Culture medium was replaced with fresh medium every 2 days. Cultures were carefully monitored daily under a heated stage inverted microscope (ECLIPSE2000-S, Nikon, Tokyo, Japan) equipped with a digital camera (Nikon, Tokyo, Japan). The cultured OSE cells were subjected to real-time polymerase chain reaction (real-time PCR).

### Histological evaluations

Isolated ovaries were fixed in 4% paraformaldehyde at 4 °C overnight, dehydrated using an ethanol series, cleared in xylene, embedded in paraffin, and sectioned at 5 μm and then stained with hematoxylin and eosin (H&E). After mounting, sections were examined under a light microscope. Follicles were counted in all sections from each ovary, and results were corrected for double counting. Follicles were classified as primordial (an oocyte surrounded by one layer of flattened granulosa cells), primary (an oocyte surrounded by one layer of cuboidal granulosa cells), secondary (two or three layers of cuboidal granulosa cells with no antral space), and antral (more than four layers of granulosa cells with one or more independent antral spaces, or with a cumulus granulosa cell layer) as previously described by Luo [30]. Follicles containing degenerated oocytes were deemed atresia based on the presence of apoptotic bodies in the granulosa cell layer, disorganized granulosa cells, a degenerating oocyte, or nuclear fragmented oocytes.

### Measurement of endocrine-related parameters and the levels of OS-associated biomarkers

Levels of serum AMH and FSH (circulating hormones) were measured using commercial ELISA kits from CUSABIO Technology LLC. Samples from each of the four animal groups were analyzed. ROS levels in serum were measured using the OxiSelect™ In vitro ROS/RNS Assay Kit (Cell Biolabs, Inc., San Diego, CA), and tissue total antioxidant capacities (T-AOCs) were determined using the Total Antioxidant Capacity assay kit (Abcam, Inc., Cambridge, UK).

### Quantitative real-time PCR

Total RNA was extracted using Trizol reagent (Invitrogen, Carlsbad, CA, USA), according to the manufacturer’s instructions. Complementary DNA was synthesized from 1 μg of total RNA using AMV Reverse Transcriptase (Promega, Madison, WI, USA) and a random hexamer (Takara Bio, Inc., Otsu, Japan) for 1 h at 42 °C, which was followed enzyme inactivation for 5 min at 95 °C. Real-time PCR was performed using TOPreal™ qPCR 2X PreMIX SYBR (Enzynomics, Daejeon, Korea). Reaction mixtures were prepared using TOPreal™ qPCR 2X PreMIX, 0.5 pmol/μl of each primer, 100 ng of cDNA, and sterile water (RNase free). The reaction conditions used were denaturation for 10 min at 95 °C, followed by 30 cycles of 10 s at 95 °C, and 30 s at 60 °C. cDNAs were subjected to PCR amplification using gene-specific primers as follows; 5′- TTGCTCCTCGTCTCTATACC − 3′ (sense) and 5′- CTAGATGGCATGGTTGG − 3′ (antisense) for Bmp-15, 5′-GAGTGTGTTGACCATTGAACC-3′ (sense) and 5′-GCACCTCGTAGCTATCATGTC-3′ (antisense) for Gdf-9, 5′-GCCTAGTCATTGTTGCA-3′ (sense) and 5′-TCCACCACCCTGTTGCTGTA-3′ (antisense) for c-Kit, 5′-AGCAGAGTGGAAAGCAACTC-3′ (sense) and 5′-CAAGCTGATTGGC GATGTGAG-3′ (antisense) for Oct-4, 5′-GCTCAAACAGGGTCTGGGAAG-3′ (sense) and 5′-GG TTGATCAGTTCTCGAGTTC3’(antisense) for MVH, and 5′-CCACAGTCCATGCCATCAC-3′ (sense) and 5′-TCCACCACCCTGTTGCTGTA (antisense) for GAPDH. Differential mRNA expressions of each target gene were calculated using the 2-ΔΔCq method and normalized versus GAPDH.

### Superovulation, zygotes collection, and embryo culture

In a separate experiment, we investigated the effect of CoQ10 on ovarian functions. Another 32 female mice were administered with CoQ10 in the same way as described above for the primary experiment, and then superovulation was induced by intraperitoneal injection with 0.1 mL of 5 IU pregnant mare’s serum gonadotropin (PMSG; Sigma-Aldrich, St Louis, MO, USA) followed by injection of 5 IU of human chorionic gonadotropin (hCG; Sigma-Aldrich) approximately 48 h later. Mice were then immediately paired 1-to-1 with 9 to 10-week-old males. The following morning mice were inspected, and those with a confirmed vaginal plug were considered fertilized. Eighteen hours after hCG injection, female mice with a confirmed vaginal plug were killed by cervical dislocation, and cumulus-enclosed one-cell embryos (zygotes) were retrieved from the ampulla portion of the oviduct and denuded by incubation for 1 min in PBS (Giboc BRL, Grand Island, NY, USA) containing 0.1% hyaluronidase (Sigma-Aldrich). Zygotes were pooled and washed three times in G-IVF-plus medium (Vitrolife, V. Frolunda, Sweden) with 10% serum substitute supplement (SSS; Irvine, Inc. Santana, USA). Healthy zygotes only were cultured under paraffin-oil in 20-μL drops of Gl-plus medium (Vitrolife) with 10% SSS for the first 2 days, and then under paraffin-oil in G2-plus medium (Vitrolife) with 10% SSS for the 2 days at 37 °C in a 5% CO_2_ incubator. Media were changed daily.

### Statistical analysis

Statistical analysis used SPSS program (version 12.0) and all data were presented as a mean ± SD. All comparisons of numbers of follicles at each developmental stage, the number of zygotes retrieved, blastocyst formation rate, and ovarian mRNA expression level for Oct-4 and MVH were analyzed by student t-test, and multiple comparisons were performed by one-way analysis of variance (ANOVA) with post hoc Bonferroni-Dunn. A *p*-value of < 0.05 was considered statistically significant.

## Results

### Effects of CoQ10 on formation of oocyte-like structure and stem cell markers expression

**Fig. 1:**
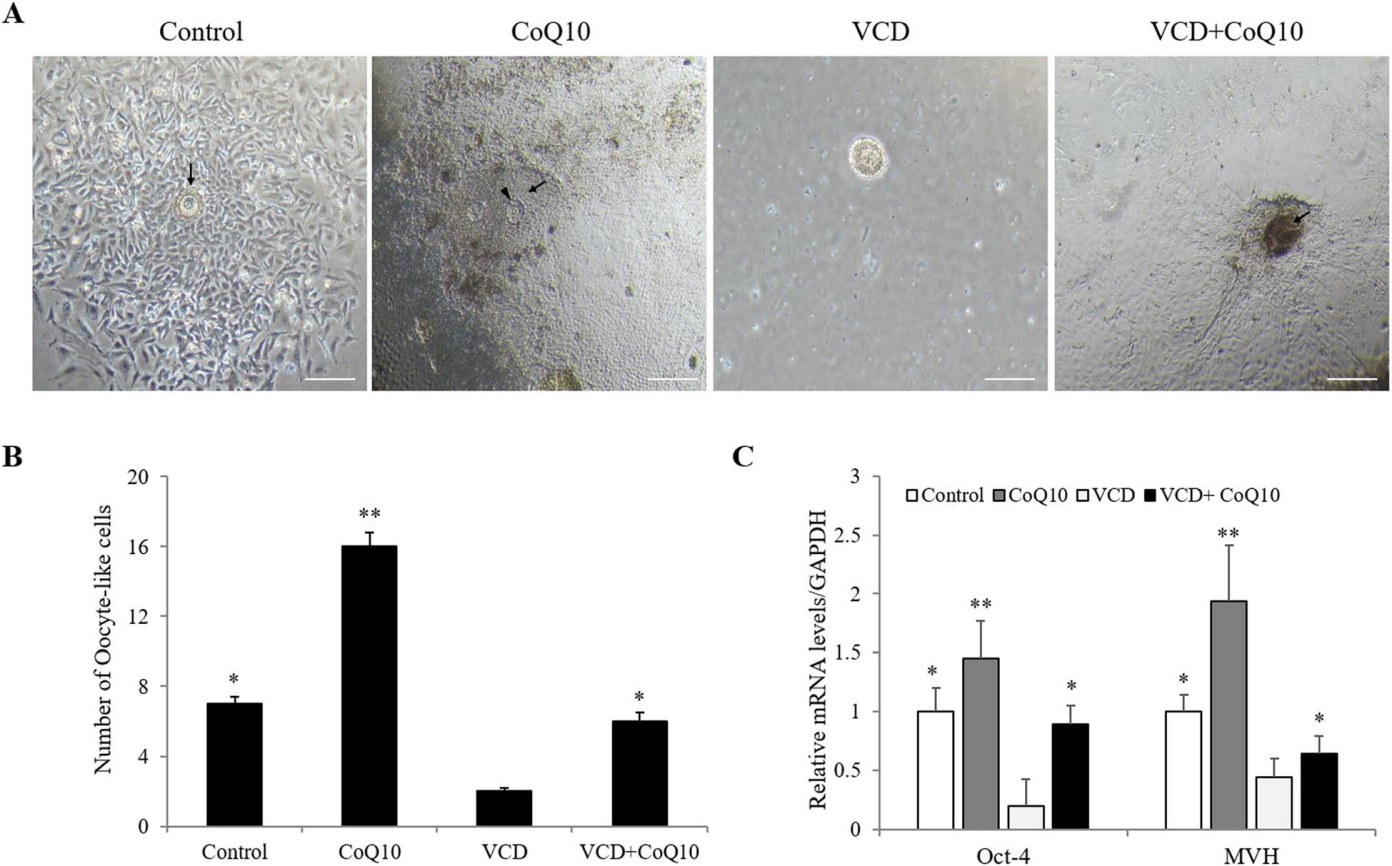
Effect of CoQ10 on oocyte-like cells formation. **a** Oocyte-like structures developed in cultured OSE obtained from mice in the study groups. **b** The numbers of in vitro differentiated oocyte-like cells. **c** Real-time PCR analysis of the mRNA expression of Oct-4 and MVH in cultured OSE. ^*^p < 0.05, ^**^p < 0.01 (versus the VCD group). Scale bar = 20 μm

Scraped OSEs were attached to the fibroblast-like appearance cells and small oocyte-like cells resembling bubble structure began to appear in close vicinity fibroblasts in all groups from about 10 to 14 days after culture of scraped OSEs. After 21 days of culture, the size of oocyte-like cells increased and oocyte-like structures with prominent nucleus (arrow head) and zona pellucida-like structure (arrow), and cytoplasmic organelles were attached to the bottom of culture dish (Fig. 1a). Numbers of oocyte-like structures were significantly smaller in the VCD group than the control group, but they were significantly greater in the VCD + CoQ10 group to the level of the control group (Fig. 1b). After 3 weeks of OSE cultures, mRNA expressions of pluripotent transcriptional marker (Oct-4) and germ cell-specific protein (mouse vasa homolog, MVH) were also significantly increased in the VCD + CoQ10 group compared to the VCD group (Fig. 1c).

### Effect of CoQ10 on ovarian function

**Fig. 2:**
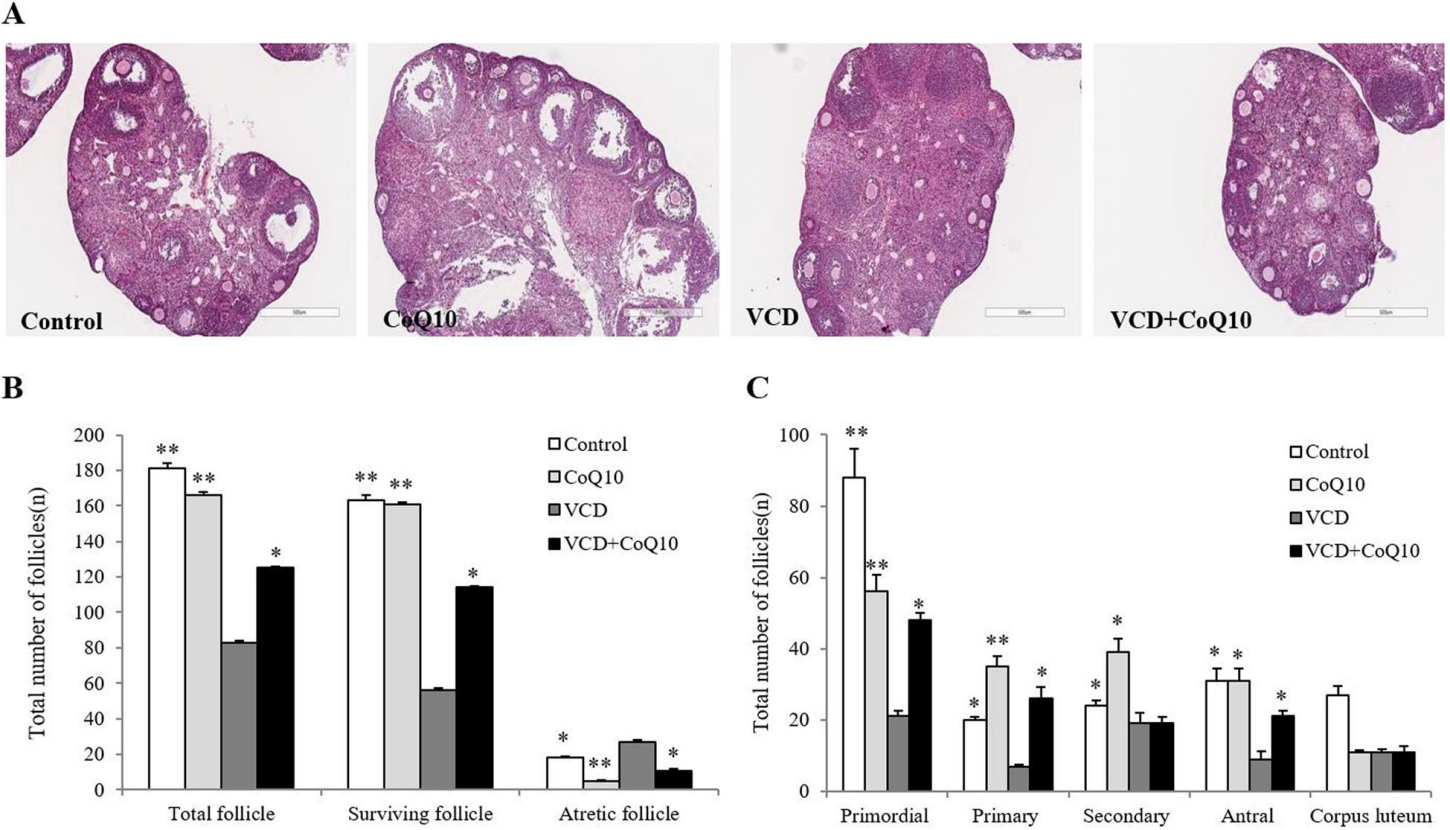
Effects of CoQ10 on follicular development. **a** Representative histological characteristics of follicles at each development stage. **b** Comparison of the total number of follicles, surviving follicles, and atretic follicles. **c** Distribution of follicles at different developmental stages. Results are presented as means±SD (*n* = 10 per each group). ^*^p < 0.05, ^**^p < 0.01 (versus the VCD group). Scale bar = 500 μm.

To investigate whether CoQ10 improves ovarian function in VCD-induced model, H&E staining was performed on ovaries. The histological characteristics of follicles at each development stage were shown in Fig. 2a. Number of surviving follicles including the primordial, primary, secondary, and antral follicles were significantly lowered in the VCD group than the control group but were significantly higher in the VCD + CoQ10 group than the VCD group. On the other hand, numbers of atretic follicles followed the order of VCD > CoQ10 + VCD > control group (Fig. 2b). The mean numbers of primordial, primary and antral follicles were 48 ± 2.2, 26 ± 3.1, and 21 ± 1.5, respectively, in the VCD + CoQ10 group, which were significantly greater than the VCD group (21 ± 1.5, 7 ± 0.5, and 9 ± 2.2, respectively). However, there was no difference in numbers of secondary follicles between the two groups (Fig. 2c).

### Effect of CoQ10 on expression of genes associated with follicular development

**Fig. 3:**
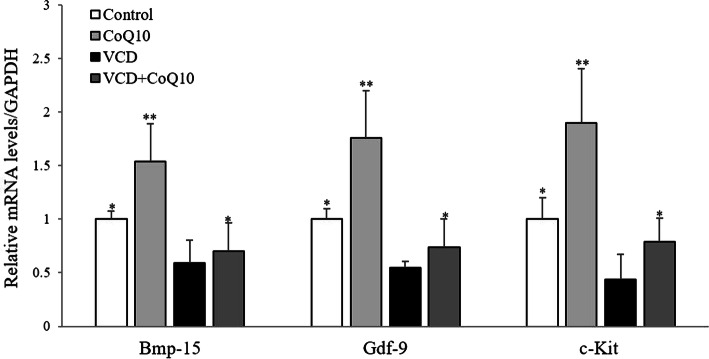
Effects of CoQ10 on ovarian mRNA expressions of Bmp-15, Gdf-9, and c-Kit. Bmp-15, Gdf-9, and c-Kit levels were normalized versus GAPDH. Results are presented as means±SD. Bmp-15, bone morphogenetic protein-15; Gdf-9, growth differentiation factor-9; GAPDH, glyceraldehyde 3-phosphate dehydrogenase. ^*^p < 0.05, ^**^p < 0.01 (versus the VCD group)

Bmp-15, Gdf-9, and c-Kit are representative factors that regulate the activation of primordial follicles and follicular development [31, 32]. The mRNA expressions of these three genes were significantly lower in the VCD group than the control group but were significantly increased in the VCD + CoQ10 group than the VCD group (*p <* 0.05) (Fig. 3).

### Effect of CoQ10 on serum hormone levels and antioxidant factor levels

**Fig. 4:**
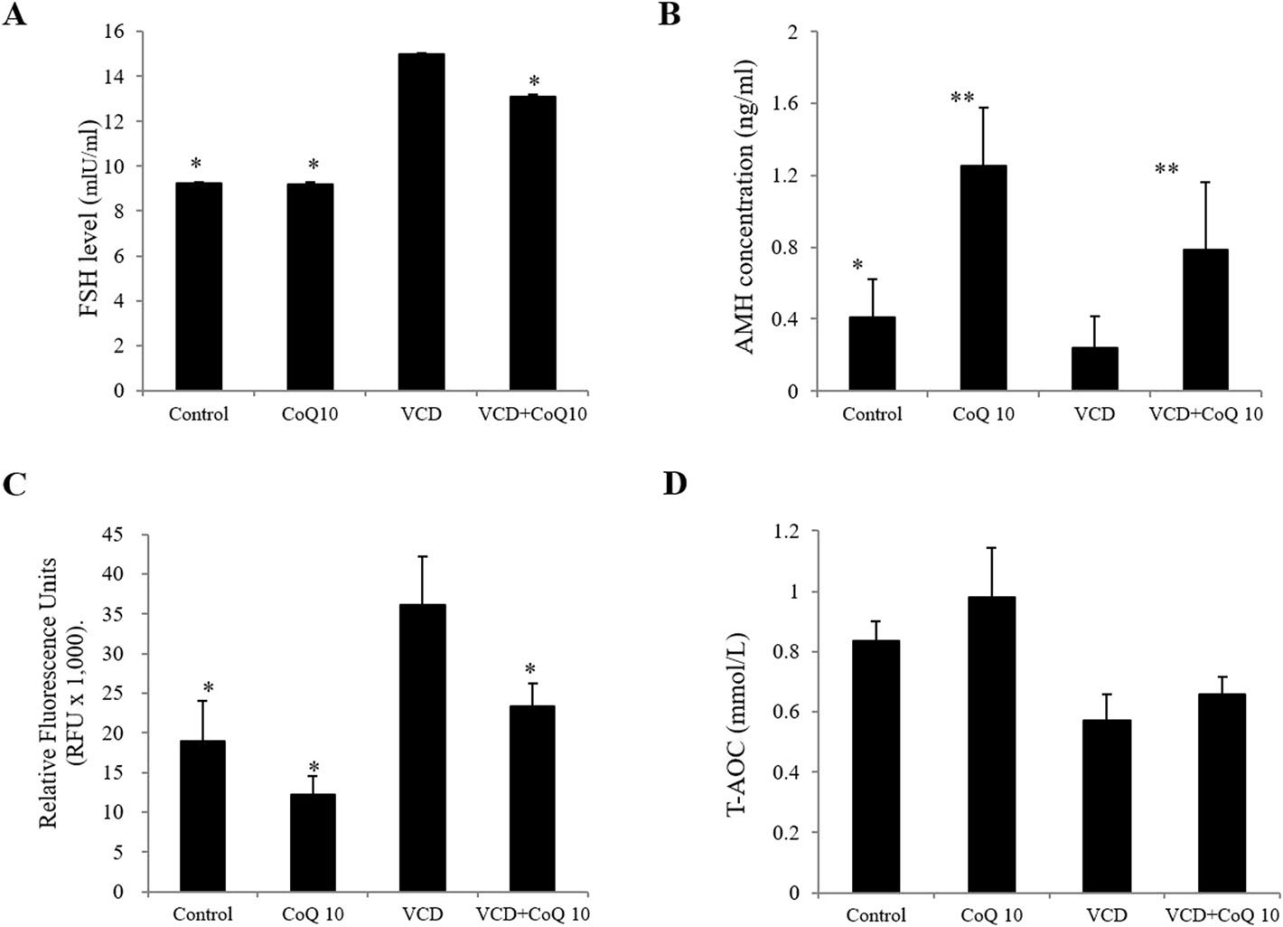
Effects of CoQ10 on serum FSH, AMH and ROS levels. **a** FSH, **b** AMH, **c** ROS. Results are presented as means±SD of four independent experiments. ^*^p < 0.05, ^**^p < 0.01 (versus the VCD group)

The mean serum FSH concentration in the control, CoQ10, VCD, and VCD + CoQ10 groups was 9.20, 9.17, 15.0, and 13.11 ng/mL, respectively (*p <* 0.01), and the mean serum FSH concentrations was significantly greater in the VCD group than the control and CoQ10 groups (*p <* 0.01). However, they were significantly lower in the VCD + CoQ10 group than in the VCD group (*p <* 0.05) (Fig. 4a).

The mean serum AMH concentrations were 0.41, 1.26, 0.24, and 0.78 ng/mL in the control, CoQ10, VCD, and VCD + CoQ10 groups, respectively (*p <* 0.01). The mean serum AMH concentration was significantly lower in the VCD group compared to the control and CoQ10 groups (*p <* 0.05 and *p <* 0.01, respectively), but was significantly higher in the VCD + CoQ10 group than in the VCD group (*p <* 0.01) (Fig. 4b). On the contrary, serum ROS level was significantly higher in the VCD group than in the control and CoQ10 groups, but it was significantly lower in the VCD + CoQ10 group than in the VCD group (Fig. 4c). T-AOC levels in ovaries were significantly lower in the VCD group than in the control groups (*p* < 0.05) and slightly higher in the VCD + CoQ10 group than in the VCD group (Fig. 4d).

### Effect of CoQ10 on ovarian response and oocyte quality

**Fig. 5:**
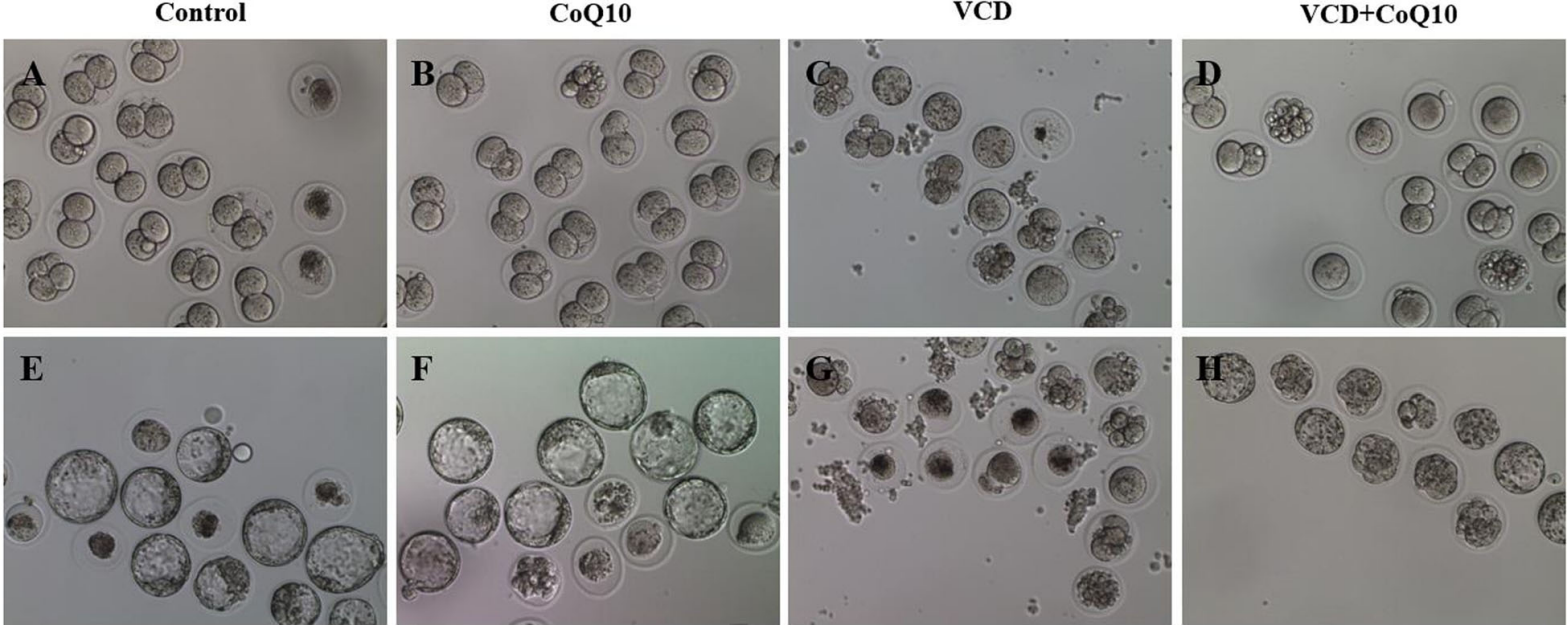
Photographs of in vitro embryo development from retrieved zygotes. **a**-**d** 2-cell embryos. **e**-**h** Blastocysts. **a**, **e** Control group. **b**, **f** the CoQ10 group. **c**, **g** the VCD group. **d**, **h** the VCD + CoQ10. Magnification × 100

In the second experiment performed to determine whether CoQ10 improves ovarian function in mice with VCD-induced ovarian failure, we assessed ovarian response and oocyte quality. Ovarian response was evaluated by counting numbers of zygotes retrieved after superovulation, followed by mating, and oocyte quality was defined as the fragmentation percentages and embryo development competency of zygotes retrieved. The number of zygotes retrieved and embryo development rate to blastocyst were both significantly lower in the VCD group than in the control group, but they were significantly higher in the VCD + CoQ10 group than in the VCD group (Fig. 5). Fragmentation percentages of retrieved zygotes was significantly greater in the VCD group than in the control group and still higher in the VCD + CoQ10 group (Table 1).

**Table 1:** Effect of CoQ10 on number of zygotes retrieved and embryo development

Group	No. of mice provided	No. of zygotes retrieved	Mean no. of zygotes retrieved /mouse	No. of zygotes fragmented (%)	No. of zygotes cultured	No. of 2-cell embryos (%)	No. of blastocysts (%)
Control	8	150	18.8 ± 2.3^*^	23 (15.3)^*^	127	90 (70.9)^*^	47 (37.0)^**^
CoQ10	8	193	24.1 ± 2.4^**^	18 (9.3)^**^	175	121 (69.1)^*^	78 (44.6)^**^
VCD	8	61	7.6 ± 2.1	21 (34.4)	40	19 (47.5)	0 (0)
VCD + CoQ10	8	108	13.5 ± 2.1^*^	24 (22.2)^*^	84	45 (53.6)	15 (17.9)^*^

^*^p < 0.05, ^**^p < 0.01 (versus VCD group)

## Discussion

Many studies have reported that CoQ10 may protect ovarian reserve against oxidative damage in mice [15–17]. Recently, some studies have attempted to confirm the potential impacts of CoQ10 on ovarian function in infertile women with low prognosis undergoing assisted reproductive technology (ART) procedures [33]. However, to the best of our knowledge, no study has yet examined the effect of CoQ10 on OSE-derived OSCs. The present study is the first to show that CoQ10 stimulates the differentiation of OSE-derived OSCs into cells with oocyte-like structure and increases the ovarian expression of Oct-4 and MVH in C57BL/6 mice with VCD-induced ovarian failure.

VCD specifically targets primordial and primary follicles and depletes follicle populations in mice and rats by accelerating atresia [34, 35]. In this respect, the VCD mouse model used provides a suitable model of ovarian failure associated with perimenopause/menopause as it induces follicle depletion but leaves ovaries intact.

In the present study, numbers of the primordial, primary and antral follicles, but not secondary follicles, were significantly greater in the VCD + CoQ10 group than in the VCD group, which suggests CoQ10 prevented follicular development inhibition of VCD. The primordial follicle to full-grown secondary follicle developmental period is preantral and independent of gonadotropin. In particular, there is considerable overlap between the intermediated primary follicle period and the initial secondary follicle period, whereas development of the fully-grown secondary to antral follicle occurs within a shorter time [36]. Thus, in this study, the reason why no difference was detected numbers of secondary follicles in the VCD and VCD + CoQ10 groups would appear to be due to overlapping of the primary and secondary follicle periods; thus, most of them were measured by the number of primary follicles.

Menopause is accompanied by ovarian failure with decreased ovarian function, which is characterized by lower serum levels of estradiol and elevated FSH level [37]. Furthermore, abnormally low FSH level and AMH level are strongly associated with pregnancy outcomes [38]. Serum AMH level is a representative marker of ovarian aging and ovarian reserve in human, and is commonly used to assess extent of ovarian follicle depletion, to diagnose premature ovarian insufficiency, and to predict age at menopause [39, 40].

In the present study, extremely high FSH levels and low AMH levels were observed in the VCD group, indicating that VCD-treated mice exhibited a typical ovarian failure. However, when VCD mice were treated with CoQ10, serum FSH levels decreased and serum AMH levels increased. AMH is synthesized by the granulosa cells of preantral and antral follicles [41], and thus, if these cells are damaged, AMH production decreases, and conversely, when these cells are recovered, the production of AMH also increases. Indeed, some studies have reported DHEA supplementation prior to assisted reproductive technology (ART) improves serum AMH levels in patients with diminished ovarian reserve [42, 43]. Özcan et al. showed that AMH concentrations in rats were reduced by cisplatin, but recovered by CoQ10 treatment [17], which suggests CoQ10 might reverse ovarian failure or aging.

In addition, a decrease or failure in ovarian function is also characterized by decrease in follicular development and oocyte quality, which determines embryo developmental competency. The results suggest that CoQ10 might restore ovarian reserve and ovarian function in mice with VCD-induced ovarian failure, which concurs with the results of several previous studies. Burstein et al. showed that CoQ10 treatment significantly improved ovarian function and mitochondrial function in 18-week-old mice, and that this resulted in an increase in ovulated oocyte numbers [44]. Ben-Meir et al. also observed that CoQ10 supplementation preserves ovarian reserve and improves mitochondrial performance and fertility in mice of reproductive age [16]. Özcan et al. introduced that CQ10 supplementation increased serum AMH level and numbers of antral follicles and that it protected ovarian reserve against oxidative damage in cisplatin-treated rats [17].

In the present study, the treatment dose of CoQ10 was determined by referring to several previous reports, in which it was administered at 150 mg/day/kg [17, 45, 46]. In this way, the present study determined the CoQ10 concentration as 150 mg/kg/day.

The present study also confirms that CoQ10 stimulates the expressions of Bmp-15, Gdf-9, and c-Kit, which are representative regulators of the activation of primordial follicles and follicular development [31, 32]. Especially, Gdf-9 and Bmp-15 play a critical role in early follicular development and ovarian function [47]. The KL/c-Kit system importantly regulates oogenesis and folliculogenesis [48, 49], which suggests that KL/c-Kit plays an important role in increasing the numbers of actively growing follicles and/or in regulating of primordial follicle maintenance.

The possible mechanism responsible for ovarian function enhancement by CoQ10 in the present study can be explained in three ways. The first involves the activation of OSCs derived from OSE and a resulting increase in ovarian reserve, and thus, in the number of follicles developing from the primordial follicle to the antral follicle increases (Fig. 2). The second involves stimulation of early follicular development, which is supported by observed increases in the expressions of Bmp-15, Gdf-9, and c-Kit (Fig. 3). A recent study also reported that administration of CoQ10 upregulated the expressions of follicle stimulating hormone receptor (FSHR) and proliferation cell nuclear antigen (PCNA), which are representative folliculogenesis-associated genes, in cyclophosphamide-induced mouse model of premature ovarian failure [50]. The third mechanism involves ROS reduction (Fig. 4c) and the stimulation of antioxidant activity by CoQ10 (Fig. 4d), which have been reported in many previous studies [17, 44, 51]. Notably, antioxidant supplementation would appear beneficial regardless of which one of these three mechanisms applies as it would reduce oxidative stress and preventor promote the repair of tissues damaged by oxidative stress [52]. Our study shows that at 150 mg/kg/day, protected female reproductive function by increasing antioxidant activity and decreasing ROS production.

Another important finding of this study is that CoQ10 contributed to protect against primary ovarian insufficiency (POI), also known as premature ovarian failure, which is a clinical syndrome defined by stop of ovarian function before the age of 40 [53]. Although the prevalence of POI is only around 1%, it is increasing due to increases in the number of premenopausal cancer survivors with iatrogenic POI due to gonadotropin treatment [54, 55]. Nevertheless, POI remains an unmet need in infertility treatment. One of the characteristics of POI is increased ROS generation, which causes ovarian tissue damage and decreases ovarian reserve [56]. Therefore, our findings suggest CoQ10 administration might protect against ovarian dysfunction in POI patients..

Improved mitochondrial function is an important requirement for the recovery of ovarian aging. However, this study did not evaluate the effect of CoQ10 treatment on mitochondrial function due to a lack of equipment. Accordingly, this is a limitation of the present study. Nevertheless, we evaluated oocyte quality, which may be a viable surrogate of mitochondrial function; thus, our results do show that oocyte quality is improved by CoQ10 treatment.

## Conclusion

The present study shows that CoQ10 stimulates the differentiation of OSE-derived OSCs and reduces ROS levels, which leads to improve follicular development, ovarian function, oocyte quality and embryo development competency in C57BL/6 mice with VCD-induced ovarian failure. These results suggest the possibility of using CoQ10 as a novel treatment strategy for ovarian aging or POI. However, further research is required to determine the optimal dosage and duration of CoQ10 supplementation.

## Data Availability

The datasets used and/or analyzed during the current study are available from the corresponding author on reasonable request.

## References

[1] May-Panloup P, Boucret L, de la Barca JM C, Desquiret-Dumas V, Ferre-L’Hotellier V, Moriniere C (2016). Ovarian ageing: the role of mitochondria in oocytes and follicles. Hum Reprod Update.

[2] Wilson DM, Sofinowski TM, McNeill DR (2003). Repair mechanisms for oxidative DNA damage. Front Biosci.

[3] Meldrum DR, Casper RF, Diez-Juan A, Simon C, Domar AD, Frydman R (2016). Aging and the environment affect gamete and embryo potential: can we intervene. Fertil Steril..

[4] Tarín JJ, Pérez-Albalá S, Cano A (2002). Oral antioxidants counteract the negative effects of female aging on oocyte quantity and quality in the mouse. Mol Reprod Dev.

[5] Katz-Jaffe MG, Lane SL, Parks JC, McCallie BR, Makloski R, Schoolcraft WB (2020). Antioxidant intervention attenuates aging-related changes in the murine ovary and oocyte. Life (Basel).

[6] Sasaki H, Hamatani T, Kamijo S, Iwai M, Kobanawa M, Ogawa S (2019). Impact of oxidative stress on age-associated decline in oocyte developmental competence. Front Endocrinol (Lausanne).

[7] Santos-Ocana C, Do TQ, Padilla S, Navas P, Clarke CF (2002). Uptake of exogenous coenzyme Q and transport to mitochondria is required for bc1 complex stability in yeast coq mutants. J Biol Chem.

[8] Villalba JM, Navas P (2000). Plasma membrane redox system in the control of stress-induced apoptosis. Antioxid Redox Signal.

[9] Pignatti C, Cocchi M, Weiss H (1980). Coenzyme Q10 levels in rat heart of different age. Biochem Exp Biol.

[10] Kalen A, Appelkvist EL, Dallner G (1989). Age-related changes in the lipid compositions of rat and human tissues. Lipids..

[11] Miles MV, Horn PS, Tang PH, Morrison JA, Miles L, DeGrauw T (2004). Age-related changes in plasma coenzyme Q10 concentrations and redox state in apparently healthy children and adults. ClinChimActa..

[12] Balercia G, Mosca F, Mantero F, Boscaro M, Mancini A, Ricciardo-Lamonica G (2004). Coenzyme Q (10) supplementation in infertile men with idiopathic asthenozoospermia: an open, uncontrolled pilot study. Fertil Steril.

[13] Bentinger M, Tekle M, Brismar K, Chojnacki T, Swiezewska E, Dallner G (2008). Stimulation of coenzyme Q synthesis. Biofactors..

[14] Mizuno K, Tanaka M, Nozaki S, Mizuma H, Ataka S, Tahara T (2008). Antifatigue effects of coenzyme Q10 during physical fatigue. Nutrition..

[15] Bentov Y, Casper RF (2013). The aging oocyte can mitochondrial function be improved?. Fertil Steril..

[16] Ben-Meir A, Burstein E, Borrego-Alvarez A, Chong J, Wong E, Yavorska T (2015). Coenzyme Q10 restores oocyte mitochondrial function and fertility during reproductive aging. Aging Cell..

[17] Özcan P, Fıçıcıoğlu C, Kizilkale O, Yesiladali M, Tok OE, Ozkan F (2016). Can coenzyme Q10 supplementation protect the ovarian reserve against oxidative damage?. J Assist Reprod Genet.

[18] Sheikhansari G, Aghebati-Maleki L, Nouri M, Jadidi-Niaragh F, Yousefi M (2018). Current approaches for the treatment of premature ovarian failure with stem cell therapy. Biomed Pharmacother.

[19] Martin JJ, Woods DC, Tilly JL (2019). Implications and current limitations of oogenesis from female germline or oogonialstem cells in adult mammalian ovaries. Cells..

[20] Wesevich V, Kellen AN, Pal L (2020). Recent advances in understanding primary ovarian insufficiency. F1000Res.

[21] Johnson J, Canning J, Kaneko T, Pru JK, Tilly JL (2004). Germline stem cells and follicular renewal in the postnatal mammalian ovary. Nature..

[22] Niikura Y, Niikura T, Tilly JL (2009). Aged mouse ovaries possess rare premeiotic germ cells that can generate oocytes following transplantation into a young host environment. Aging..

[23] Zhang Y, Yang Z, Yang Y, Wang S, Shi L, Xie W (2011). Production of transgenic mice by random recombination of targeted genes in female germline stem cells. J Mol Cell Biol.

[24] White YAR, Woods DC, Takai Y, Ishihara O, Seki H, Tilly JL (2012). Oocyte formation by mitotically active germ cells purified from ovaries of reproductive-age women. Nat Med.

[25] Zou K, Yuan Z, Yang Z, Luo H, Sun K, Zhou L (2009). Production of offspring from a germline stem cell line derived from neonatal ovaries. Nat Cell Biol.

[26] Xu J, Zheng T, Hong W, Ye H, Hu C, Zheng Y (2018). Mechanism for the decision of ovarian surface epithelial stem cells to undergo neo-oogenesis or ovarian tumorigenesis. Cell Physiol Biochem.

[27] Bukovsky A, Copas P, Virant-Klun I (2006). Potential new strategies for the treatment of ovarian infertility and degenerative diseases with autologous ovarian stem cells. Expert Opin Biol Ther.

[28] Virant-Klun I (2015). Postnatal oogenesis in humans: a review of recent findings. Stem Cells Cloning.

[29] Silvestris E, Cafforio P, D'Oronzo S, Felici C, Silvestris F, Loverro G (2018). In vitro differentiation of human oocyte-like cells from oogonial stem cells: single-cell isolation and molecular characterization. Hum Reprod.

[30] Luo LL, Huang J, Fu YC (2008). Effects of tea polyphenols on ovarian development in rats. J Endocrinol Investig.

[31] Paulini F, Melo EO (2011). The role of oocyte-secreted factors GDF9 and BMP15 in follicular development and oogenesis. Reprod Domest Anim.

[32] Hsueh AJ, Kawamura K, Cheng Y, Fauser BC (2015). Intraovarian control of early folliculogenesis. Endocr Rev.

[33] Florou P, Anagnostis P, Theocharis P, Chourdakis M, Goulis DG (2020). Does coenzyme Q10 supplementation improve fertility outcomes in women undergoing assisted reproductive technology procedures? A systematic review and meta-analysis of randomized-controlled trials. J Assist Reprod Genet.

[34] Springer LN, McAsey ME, Flaws JA, Tilly JL, Sipes IG, Hoyer PB (1996). Involvement of apoptosis in 4-vinylcyclohexene diepoxide-induced ovotoxicity in rats. Toxicol Appl Pharmacol.

[35] Brooks HL, Pollow DP, Hoyer PB (2016). The VCD mouse model of menopause and perimenopause for the study of sex differences in cardiovascular disease and the metabolic syndrome. Physiology (Bethesda).

[36] Klein NA, Harper AJ, Houmard BS, Sluss PM, Soules MR (2002). Is the short follicular phase in older women secondary to advanced or accelerated dominant follicle development?. J Clin Endocrinol Metab.

[37] Powell CM, Taggart RT, Drumheller TC, Wangsa D, Qian C, Nelson LM (1994). Molecular and cytogenetic studies of an X;autosome translocation in a patient with premature ovarian failure and review of the literature. Am J Med Genet.

[38] Chen YP, Wu WH, Wu HM, Chen CK, Wang HS, Huang HY (2014). Effects of anti-Müllerian hormone and follicle stimulating hormone levels on in vitro fertilization pregnancy rate. Taiwan J Obstet Gynecol.

[39] Kallio S, Aittomaki K, Piltonen T, Veijola R, Liakka A, Vaskivuo TE (2012). Anti-Mullerian hormone as a predictor of follicular reserve in ovarian insufficiency: special emphasis on FSH-resistant ovaries. Hum Reprod.

[40] Dolleman M, Depmann M, Eijkemans MJ, Heimensem J, Broer SL, van der Stroom EM (2014). Anti-Mullerian hormone is a more accurate predictor of individual time to menopause than mother’s age at menopause. Hum Reprod.

[41] Dewailly D, Andersen CY, Balen A, Broekmans F, Dilaver N, Fanchin R (2014). The physiology and clinical utility of anti-Mullerian hormone in women. Hum Reprod Update.

[42] Yilmaz N, Uygur D, Inal H, Gorkem U, Cicek N, Mollamahmutoglu L (2013). Dehydroepiandrosterone supplementation improves predictive markers for diminished ovarian reserve: serum AMH, inhibin band antral follicle count. Eur J Obstet Gynecol Reprod Biol.

[43] Singh N, Zangmo R, Kumar S, Roy KK, Sharma JB, Malhotra N, et al. A prospective study on roleof dehydroepiandrosterone (DHEA) on improving theovarian reserve markers in infertile patients withpoor ovarian reserve. Gynecol Endocrinol. 2013;29:989–92. 10.3109/09513590.2013.824957 24004296

[44] Burstein E, Perumalsamy A, Bentov Y, Esfandiari N, Jurisicova A, Casper RF (2009). Co-enzyme Q10 supplementation improves ovarian response and mitochondrial function in aged mice. Fertil Steril.

[45] Zhang YP, Eber A, Yuan Y, Yang Z, Rodriguez Y, Levitt RC, et al. Prophylactic and antinociceptive effects of coenzyme Q10 on diabetic neuropathic pain in a mouse model of type 1 diabetes. Anesthesiology. 2013;118:945–54. 10.1097/ALN.0b013e3182829b7b 23334664

[46] Garrido-Maraver J, Cordero MD, Oropesa-Ávila M, Fernández Vega A, de la Mata M, Delgado Pavón A, et al. Coenzyme q10 therapy. Mol Syndromol. 2014;5:187–97. 10.1159/000360101 PMC4112525 25126052

[47] Chang HM, Qiao J, Leung PC (2016). Oocyte-somatic cell interactions in the human ovary novel role of bone morphogenetic proteins and growth differentiation factors. Hum Reprod Update.

[48] Driancourt MA, Reynaud K, Cortvrindt R, Smitz J (2000). Roles of KIT and KIT LIGAND in ovarian function. Rev Reprod.

[49] Hutt KJ, McLaughlin EA, Holland MK (2006). Kit ligand and c-kit have diverse roles during mammalian oogenesis and folliculogenesis. Mol Hum Reprod.

[50] Delkhosh A, Delashoub M, Tehrani AA, Bahrami AM, Niazi V, Shoorei H (2019). Upregulation of FSHR and PCNA by administration of coenzyme Q10 on cyclophosphamide-induced premature ovarian failure in a mouse model. J Bio Chem Mol Toxicol.

[51] Quinzii CM, Tadesse S, Naini A, Hirano M (2012). Effects of inhibiting CoQ10 biosynthesis with 4-nitrobenzoate in human fibroblasts. PLoS One.

[52] Rajaei S, AlihemmatiPh DA, AbedelahiPh DA (2019). Antioxidant effect of genistein on ovarian tissue morphology, oxidant and antioxidant activity in rats with induced polycystic ovary syndrome. Int J Reprod Biomed.

[53] Webber L, Davies M, Anderson R, Bartlett J, Braat D (2016). ESHRE guideline: management of women with premature ovarian insufficiency. Hum Reprod.

[54] van Dorp W, Mulder RL, Kremer LCM, Hudson MM, van den Heuvel-Eibrink MM, van den Berg MH (2016). Recommendations for premature ovarian insufficiency surveillance for female survivors of childhood, adolescent, and young adult Cancer: a report from the international late effects of childhood cancer guideline harmonization Group in Collaboration with the pan care sur fup consortium. J Clin Oncol.

[55] Béranger R, Hoffmann P, Christin-Maitre S, Bonneterre V (2012). Occupational exposures to chemicals as a possible etiology in premature ovarian failure: a critical analysis of the literature. Reprod Toxicol.

[56] Jeelani R, Khan SN, Shaeib F, Kohan-Ghadr HR, Aldhaheri SR, Najafi T (2017). Cyclophosphamide and acrolein induced oxidative stress leading to deterioration of metaphase II mouse oocyte quality. Free Radic Biol Med.

